# Successful monozygotic triplet pregnancy after a single blastocyst transfer following in vitro maturation of oocytes from a woman with polycystic ovary syndrome: a case report

**DOI:** 10.1186/s12884-020-2750-4

**Published:** 2020-01-29

**Authors:** Kuniaki Ota, Toshifumi Takahashi, Mikiko Katagiri, Ryu Matsuoka, Akihiko Sekizawa, Hideki Mizunuma, Hiroaki Yoshida

**Affiliations:** 1 Fukushima Medical Center for Children and Women, Fukushima Medical University, 1 Hikarigaoka, Fukushima City, Fukushima 960-1295 Japan; 2 Sendai ART Clinic, 206-13 Nagakecho, Miyagino, Sendai, Myagi 983-0864 Japan; 3 Department of obstetrics and gynecology, Showa University, 1-5-8 Hatanodai, Shinagawa, Tokyo, 142-8555 Japan

**Keywords:** Assisted reproductive technology, In vitro maturation of oocyte, monozygotic triplets, polycystic ovary syndrome, Zygotic splitting

## Abstract

**Background:**

Although women with polycystic ovarian syndrome (PCOS)-related sub-fertility are high responders to controlled ovarian stimulation, it is difficult to obtain mature oocytes in these women. Therefore, in vitro maturation (IVM), which is the technique of letting the contents of the ovarian follicles and the oocytes inside mature in vitro, has often been proposed in such women. We describe the first successful delivery of monozygotic triplets resulting from transfer of a single blastocyst following IVM of oocytes.

**Case presentation:**

A 32-year-old nulligravida female with PCOS underwent IVM. She underwent vitrified-warmed single blastocyst transfer following IVM, and a dichorionic triamniotic triplet pregnancy was confirmed at 8 weeks. Healthy triplets were delivered by cesarean section at 33 weeks’ gestation. This is the first case of monozygotic triplets derived from IVM oocytes that were successfully delivered. The determination of chorionicity and amnionicity is generally supposed until 3 days after fertilization, and no division or splitting of her embryo was observed on transfer. Interestingly, her embryo might have split after the transfer, resulting in a dichorionic triamniotic triplet pregnancy.

**Conclusions:**

Patients should be informed of a possible increased risk of monozygotic multiple pregnancies after single embryo transfer following IVM.

## Background

However, even if the single embryo transfer (SET) is performed, the multiple pregnancies, such as twin or more pregnancies, has been reported [1–3]. So far, there is evidence that the rate of monozygotic multiple pregnancies by assisted reproductive technology (ART) (1.01–2.24%) is higher than those by spontaneous conception (0.40–0.45%) [4–7]. Although the precise mechanisms of higher rate of monozygotic splitting by ART treatment remain unknown, there have been reported about risk factors for monozygotic splitting, such as zona or oocyte membrane breaking for intracytoplasmic sperm injection (ICSI), embryo biopsy, and assisted hatching, and extended culture period for blastocyst-stage embryo culture [1–3, 8, 9].

In vitro maturation (IVM) of immature oocytes is a promising method for collecting eggs without side effects, such as ovarian hyperstimulation syndrome (OHSS), in polycystic ovary syndrome (PCOS) women [10]. Many cases of pregnancy and delivery of healthy children by ART treatments using oocytes derived from IVM of immature oocytes in PCOS women [11]. To date, the combined technique of cryopreservation and IVM is established treatment to prevent OHSS in PCOS women [12, 13] and this strategy obtains a pregnancy rate of up to 35–40% [14]. However, this method assumes various unclear risks associated with long-term culture.

There have been some reports that SET following IVM of immature oocytes retrieved from infertile woman resulted in twin pregnancy [15, 16], but it has not been clarified in IVM whether a longer culture time may cause zygotic splitting. Nevertheless, the actual number of births from IVM is not clear because IVM is performed in only a limited number of institutes worldwide. Herein, we describe our first experience with the first successful delivery of monozygotic triplets resulting from single blastocyst transfer following IVM of oocytes.

## Case presentation

A 32-year-old nulligravida Asian female visited the Sendai Art Clinic with primary infertility, including anovulation for the past 2 years. She was diagnosed with PCOS according to the Rotterdam ESHRE/ASRM consensus criteria [17]. Hysterosalpingography showed bilateral tubal patency and no defects of uterine cavity. Her husband’s semen analysis showed normozoospermia, based on the 2010 World Health Organization criteria. After she had failed to achieve pregnancy following several courses of ovulation induction with clomiphene citrate or gonadotrophin injections, she opted for IVM treatment to retrieve germinal vesicle (GV) oocytes for in vitro culture. Informed consent was provided.

She was initiated on an IVM-in vitro fertilization (IVF) protocol on day 6 of a spontaneous menstrual cycle and given 4 days of 75 IU recombinant FSH (Gonal-F, Serono Japan, Tokyo, Japan). She was repetitively evaluated on days 6 and 8 of the menstrual cycle by transvaginal ultrasound scans. When the leading follicle size was less than 12 mm, 5000 IU of urinary human chorionic gonadotropin (hCG) (gonatropin, ASKA Pharmaceutical Co., Ltd., Tokyo, Japan) was administered and oocyte retrieval was carried out 34 h after hCG triggering. The collection and culture of immature oocytes were performed as previously described [15].

**Fig. 1:**
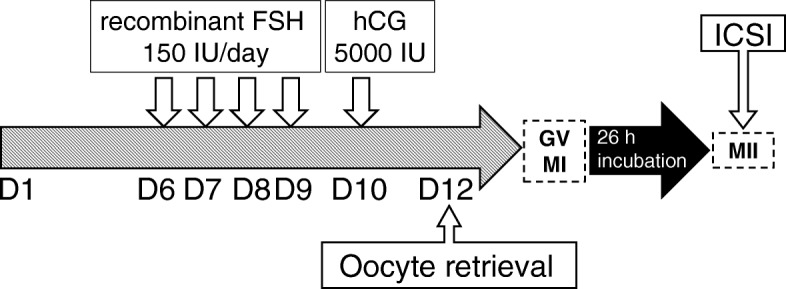
Time course of the invitro maturation-in vitro fertilization protocol. FSH: follicle stimulating hormone, hCG: human chorionic gonadotropin, GV: germinal vesicle, MI: metaphase I, MII: metaphase II, ICSI: intracytoplasmic sperm injection

Fifteen GV stage immature oocytes were retrieved and incubated for 26 h in IVM culture medium (MediCult IVM medium, ORIGIO Japan, Yokohama, Japan) supplemented with recombinant FSH 75 mIU/ml (Gonal-F, Serono Japan, Tokyo, Japan), hCG 100 mIU/ml (gonatropin, ASKA Pharmaceutical Co., Ltd., Tokyo, Japan), and 10% patient’s inactivated serum. The oocytes were then denuded from the cumulus cells. Eleven mature oocytes were obtained and subjected to ICSI with fresh sperm (Fig. 1). Zygotes were cultured in culture medium (Universal IVF, ORIGIO Japan, Yokohama, Japan), and fertilization was judged 16–18 h after ICSI for the appearance of two distinct pronuclei and two polar bodies. Nine (81.8%) oocytes were fertilized. Fertilized zygotes were transferred into culture medium (IVC-1, In Vitro Care, MD, Frederick, USA) and all embryos developed to blastocysts on day 5 (one middle expanding blastocyst and early blastocysts) and were cryopreserved by vitrification.

**Fig. 2:**
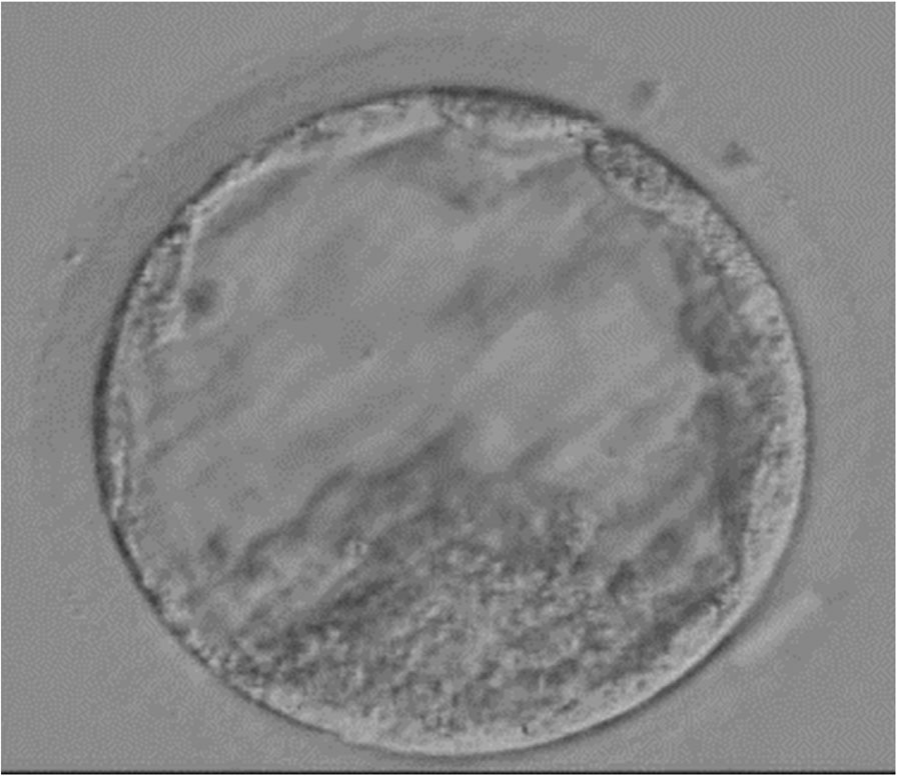
Vitrified-warmed blastocyst graded to 4 BC according to Gardener’s classification

**Fig. 3:**
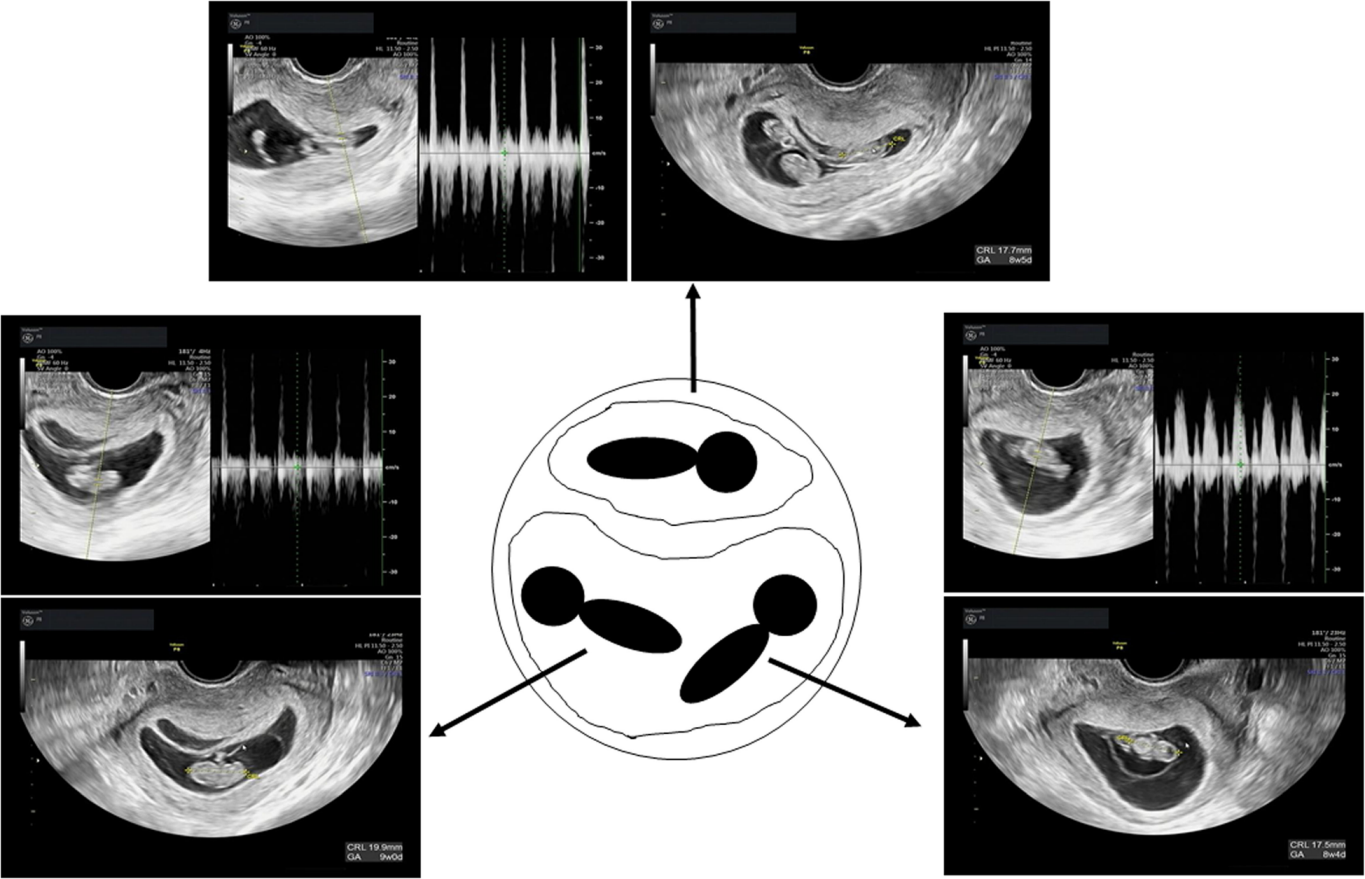
Representative images of transvaginal echocardiography at 8 weeks and 4 days. Three fetuses with heartbeats in the two gestational sacs were observed

For the preparation of the endometrium, transcutaneous estradiol (Estrana TAPE 0.72 mg, Hisamitsu Pharmaceutical, Tokyo, Japan; four sheets every 2 days) was administered on day of the hormone replacement therapy (HRT). The endometrial lining was evaluated on days 12–14 of the HRT cycle and the endometrial thickness needed to be a minimum of 8 mm. A vaginal progesterone suppository (Luteum, Aska pharmacy, Tokyo, Japan; 2 tablets everyday) was administered when the endometrial thickness achieved was greater than 8 mm. The transcutaneous estradiol and vaginal progesterone suppository were continued until 9 weeks of gestation. A single frozen-thawed blastocyst (Gardner’s classification:4 BC) (Fig. 2) was transferred under transvaginal ultrasound guidance on day 5 after vaginal progesterone suppository was administered. Two weeks after blastocyst transfer, the patient’s serum hCG concentration was 2194 IU/L and pregnancy was confirmed. Transvaginal ultrasound performed at 5 weeks and 0 days of gestation showed 2 gestational sacs inside the uterus. At 8 weeks 2 days gestation, 3 fetal heart beats were detected and a dichorionic triamniotic triplet pregnancy was observed by ultrasound (Fig. 3). The subsequent pregnancy course went well until 28 weeks 5 days when she was admitted to the maternal fetus intensive care unit for fetal growth restriction. She received prenatal steroids for fetal lung maturation and intravenous magnesium sulfate for tocolysis until the delivery. At 33 weeks 4 days, three baby girls were delivered by an elective Caesarean section. Their weight and APGAR scores at 1 min and 5 min were 1852 g, 8–8, 1817 g, 7–8, and 1495 g, 8–9, respectively. Histological examination of the placenta confirmed monochorionicity. The babies remained in the neonatology unit without morbidity and were discharged 4 weeks after birth.

## Discussion and conclusions

Here, we describe a case of a triplet pregnancy from a SET following IVM with a successful delivery at 33 weeks. This triplet pregnancy was considered dichorionic derived from monozygotic embryos following IVM. This patient was diagnosed with anovulatory PCOS, and therefore, a concurrent spontaneous pregnancy at the same time as SET was not considered. Therefore, this pregnancy was considered monozygotic triplets and, moreover, the gender was the same female. To the best of our knowledge, this is the first report on a successful delivery of monozygotic triplets after SET following IVM.

A dichorionic twins occurs when an embryo splits until 3 days after fertilization and a monochorionic twins occurs when an embryo splits 4–8 days after fertilization [18]. These phenomena are speculated based on natural pregnancy. In the ART treatment cycles, dichorionic twins originated from SET have been reported [19, 20]. This does not fit the theory of embryo splitting timing and forming monozygotic dichorionic twins. In the present case, none of the embryo split was observed at the time of transfer 5 days after fertilization. There is a possibility that the embryo split after the transfer and became a dichorionic triamniotic triplet.

Very recently, Ikemoto et al. reported that the prevalence of a triplet pregnancy after SET following IVF and ICSI was 0.04% in the Japanese ART data [21]. However, their data cannot distinguish a triplet pregnancy following IVM. On the other hand, due to the small number of babies born following IVM worldwide [22, 23], it is not possible to mention the relationship between IVM and monozygotic triplets.

There is currently little data on the mechanisms of zygotic splitting leading to monozygotic multiple pregnancies. Several factors have been discussed in the literature but not easy to take off form the hypothesis. Generally, possible risk factors for the increasing rate of zygotic splitting has been speculated prolonged culturing of embryos and zona pellucida manipulation, such as assisted hatching, ICSI, and embryo biopsy [24–28]. IVM may increase the occurrence of zygote splitting due to longer in vitro culturing before insemination. In fact, in the present case, embryos were cultured in vitro for 6 days (1 day before fertilization and 5 days after ICSI). Therefore, the extension of the culture time was one of the most speculated cause for zygotic splitting. However, at present, the cause of zygotic splitting remains unknown. The patient’s genetic predisposition, including PCOS itself, the effects of drugs administered before oocytes collection, and the effects of in vitro maturation culture may also be related to zygotic splitting [29].

In conclusion, we report here the first case of a monozygotic triplet pregnancy from IVM oocytes with a successful delivery after intensive perinatal care. Patients should be informed of a possible increased risk of monozygotic multiple pregnancies after SET. The frequency and mechanism of how the monozygotic triplet pregnancy occurs during the IVM is still unknown. Future studies should be needed to clarify the relationship between zygotic splitting and IVM and other ART procedures.

## Data Availability

The datasets during and/or analyzed during the current study available from the corresponding author on reasonable request.

## Abbreviations

ART: Assisted reproductive technology
ET: Embryo transfer
GV: Germinal vesicle
hCG: human chorionic gonadotropin
HRT: Hormone replacement therapy
ICSI: Intracytoplasmic sperm injection
IVF: In vitro fertilization
IVM: In vitro maturation
OHSS: Ovarian hyperstimulation syndrome
PCOS: Polycystic ovary syndrome
SET: Single embryo transfer
